# Prolonged treatment with a PI3K p110α inhibitor causes sex- and tissue-dependent changes in antioxidant content, but does not affect mitochondrial function

**DOI:** 10.1042/BSR20201128

**Published:** 2020-10-16

**Authors:** Christopher P. Hedges, Toan Pham, Bhoopika Shetty, Stewart W.C. Masson, Anthony J.R. Hickey, Peter R. Shepherd, Troy L. Merry

**Affiliations:** 1 Discipline of Nutrition, School of Medical Sciences, University of Auckland, Auckland, New Zealand; 2 Maurice Wilkins Centre for Molecular Biodiscovery, University of Auckland, Auckland, New Zealand; 3 Applied Surgery and Metabolism Laboratory, School of Biological Sciences, University of Auckland, Auckland, New Zealand; 4 Molecular Medicine and Pathology, School of Medical Sciences, The University of Auckland, Auckland, New Zealand

**Keywords:** BYL-719, free radicals, mitochondrial respiration, oxidative stress, phosphoinositide 3-kinase

## Abstract

Genetic inhibition of the p110α isoform of phosphatidylinositol-3-kinase (PI3K) can increase murine lifespan, enhance mitochondrial function and alter tissue-specific oxidative balance. Here, we investigated whether pharmacological inhibition of the p110α isoform of PI3K induces similar enhancement of mitochondrial function in middle-aged mice. Eight-month-old male and female mice were fed a diet containing 0.3 g/kg of the p110α-selective inhibitor BYL-719 (BYL) or a vehicle diet (VEH) for 6 weeks. Mice consuming BYL-719 had higher blood glucose and insulin, and tended towards decreased body weight. After 72 h, gene expression of the mitochondrial biogenesis mediators *Pgc1α, Tfam* and *Nrf1* was greater in liver of BYL-719 males only, but unchanged in skeletal muscle of either sex. Six weeks of BYL-719 treatment did not affect mitochondrial content or function in the liver or skeletal muscle of either sex. In livers of males only, the expression of the antioxidant genes *Nfe2l2, Cat, Sod1* and *Sod2* increased within 72 h of BYL-719 treatment, and remained higher after 6 weeks. This was associated with an increase in hepatic GSH content and catalase protein expression, and lower H_2_O_2_ levels. Our results suggest that pharmacological inhibition of p110α in adult mice does not affect liver or skeletal muscle mitochondrial function, but does show sex- and tissue-specific effects on up-regulation of antioxidant response.

## Introduction

Mitochondria play a central role in coordinating cellular homoeostasis [1], and are putative mediators of health and disease, with mitochondrial dysfunction associated with the ageing process [2,3], and the development of many disease states [4–8]. Strategies that enhance mitochondrial efficacy and capacity are therefore desirable to prolong life- and health-span. Mice which lack the insulin receptor in adipose tissue showed increased lifespan [9], and this is associated with increased mitochondrial gene expression and oxidative metabolism [10]. Similarly, heterozygous mutation of phosphatidylinositol-3-kinase (PI3K), which renders the p110α isoform catalytically inactive, results in mice that display normal glucose homoeostasis and insulin sensitivity with age, and increased lifespan [11]. Importantly, mice homozygous for this mutation are non-viable [12], providing evidence that while suppressing p110α activity does not result in an overt diabetic phenotype, some residual activity is required to maintain cellular homoeostasis.

Skeletal muscle is a metabolically dynamic tissue that is rich in mitochondria, and functionally declines markedly with ageing and metabolic disease [13–16]. Muscle-specific knockout of p110α enhances mitochondrial oxidative capacity and increases reactive oxygen species (ROS) production [17]. The theory of mitohormesis proposes that ROS, primarily those produced by mitochondria, contribute to progressive damage to proteins and DNA, ultimately leading to the deterioration of cellular function [18,19], but that transient bursts in ROS can promote antioxidant adaptations that extend lifespan [20–22]. Thus, an increase in ROS production in muscle-specific knockout p110α is consistent with an increased lifespan in whole-body p110α knockdown. Collectively, this suggests that specific down-regulation of the p110α isoform of PI3K may induce mitochondrial and antioxidant-defence system adaptations, with the potential to extend life- and health-span, without dramatically impairing blood glucose regulation. Recapitulating the effects of these genetic modifications via a pharmacological inhibitor presents a potential avenue for promoting health in humans.

Oncogenic mutations in PI3K has led to the development of isoform-specific pharmacological inhibitors of PI3K that are currently approved for clinical trials, and we have previously shown that daily i.p. administration of the PI3K p110α isoform inhibitor A66 is tolerated by young (4–5 weeks old) mice, but may lead to developmental defects in bone strength, and reduce fat and body mass gain, without altering insulin sensitivity [23]. Therefore, to assess whether pharmacological PI3K inhibition has the potential to promote health span, we sought to determine the effect of prolonged oral treatment with the selective PI3K p110α inhibitor BYL-719 on liver and skeletal muscle mitochondrial function and content, and oxidative stress, in adult mice. We report here that despite acutely elevating hepatic mitochondrial biogenesis-related genes in the liver of male mice, 6 weeks of treatment with the PI3K p110α inhibitor BYL-719 did not enhance mitochondrial function, but did alter antioxidant pathways.

## Methods

### PI3K inhibition in mice

The experimental diet was made in-house by blending a standard chow diet (Teklad Global 2018, Harlan Laboratories, Madison, WI, U.S.A.), which was then mixed with water containing either BYL-719 (MedChem Express) dissolved in DMSO, or DMSO as a vehicle-only control. The final concentration of DMSO in both diets was 3.75 ml DMSO per kg food. Once mixed, diets were dehydrated overnight, which was confirmed by weighing diet prior to incorporation of either BYL-719 or vehicle solution, and again after dehydration. To determine an orally bioavailable study dose, pilot experiments were conducted using doses of 0.15, 0.30 and 0.60 g/kg BYL-719, which 6-month-old male mice were fed *ad libitum* for 1 week, after which blood glucose and plasma insulin were measured. From this, we selected a dose of 0.3 g/kg BYL719 as a study dose to achieve mild hyperglycemia.

Following this pilot study, adult (8-month-old) male and female C57Bl6/J mice were obtained from the AgResearch Small Animal Colony (Ruakura, New Zealand), and experiments were conducted at the University of Auckland. Mice were housed in groups of three to five in Individually Ventilated Cages (Techniplast GM500 Mouse IVC Green Line). Littermates were block-randomised to receive either the diet containing either 0.3 g/kg BYL-719 (BYL) or vehicle (VEH) for either 72 h (*n*=10 in all groups) or 6 weeks (*n*=12 males in each group, *n*=15 females in each group). Animals had access to tap water and experimental diet *ad libitum*, and body mass and food intake per cage were measured weekly. All animal experiments were approved by the University of Auckland Animal Ethics Committee (R001960).

### Treadmill performance

Time to fatigue and distance run was determined using a motorised treadmill (Panlab/Harvard Apparatus, Holliston, MA, U.S.A.) after 5 weeks of BYL-719 consumption. As described previously [24], mice were familiarised with the treadmill by exposure to speeds up to 10 cm/s for a total of 5 min, completed twice on non-consecutive days. To assess exercise performance, the treadmill was set at an initial speed of 10 cm/s, and increased 4 cm/s every 3 min until exhaustion, with no slope (0°). Mice were motivated to run by a shock grid at the rear of the belt, which delivered a current of 0.3 mA. Exhaustion was determined as the point when mice remained on the shock grid for three consecutive shocks.

### Tissue and blood collection and preparation

Following the 6-week intervention period, blood samples were collected from a tail vein following an overnight fast (fasted) and when re-fed *ad libitum* for 1 h (fed). Blood glucose was determined using a hand-held glucose meter (Accu-Chek Performa; Roche, Basel, Switzerland) and plasma insulin with AlphaLISA immunoassay detection kit (PerkinElmer, Waltham, MA). Mice were killed by cervical dislocation and a small portion of liver and skeletal muscle (gastrocnemius) was placed in organ transplant buffer (custodiol HTK) to measure mitochondrial function, with the remaining tissue being snap-frozen in liquid nitrogen and stored at −80°C until analysis.

### Mitochondrial respiration and ROS emission

Mitochondrial respiration was measured in duplicate from permeabilised skeletal muscle fibres (∼2 mg) and from liver homogenate (equivalent to 2.5 mg wet weight), as previously described [25,26]. Mitochondrial respiration was measured in a range of states representing non-phosphorylating (leak), phosphorylating (oxidative phosphorylation, oxphos), and uncoupled respiration, from both skeletal muscle and liver. Complex I (CI) leak respiration was measured with the addition of malate (2 mM) and pyruvate (5 mM), following which ADP (2.5 mM) was added to measure CI oxphos. Succinate was then added to measure oxphos supported by complex I and II substrates (CI and II oxphos). Oligomycin was then added to inhibit ATP synthase to measure CI and II leak respiration. Stepwise titrations of 0.5 µM CCCP were then added to determine maximal uncoupled respiration, before respiration was inhibited at complex III (CIII) with antimycin a. Finally, Complex IV (cytochrome *c* oxidase, CCO) activity was measured using ascorbate (2 mM) and TMPD (0.5 mM), following which CIV was inhibited with sodium azide (100 mM). All respiration rates were corrected for non-mitochondrial oxygen consumption using the oxygen consumption rate determined following inhibition of CIII, and complex IV respiration rate was corrected for auto-oxidation in each assay.

H_2_O_2_ emission was determined from skeletal muscle simultaneously with respiration using integrated spectrofluorimeters. Exogenous superoxide dismutase (SOD, 10 U) and horseradish peroxidase (HRP, 5 U) were added to oxygraph chambers, along with Amplex Ultra Red (25 µM, Thermo Fisher Scientific, Waltham, MA, U.S.A.). All mitochondrial respiration rates and ROS emission were recorded and quantified using Datlab 7.1 software (Oroboros Instruments).

### Gene expression analyses

mRNA was isolated from frozen liver and muscle samples using Quick-RNA™ MiniPrep Plus (Zymo Research, California, U.S.A.) following manufacturer’s instructions and mRNA was reverse-transcribed using a high-capacity cDNA reverse transcription kit (Applied Biosystems, Warrington, U.K.). Quantitative PCR (qPCR) was used to assess the expression of *Pgc1a, Tfam, Cox4, Nrf1, Nfe2l2, Cat, Sod1* and *Sod2* mRNA in triplicate using a QuantStudio 6 flex system (Applied Biosystems) with SYBR® Select Master Mix (Applied Biosystems), and custom primers (Integrated DNA Technologies, Iowa, U.S.A.). Primer sequences used are found in Table 1. Primer specificity was verified by melt curve analysis and in all cases detected a single peak. Expression of mRNA was normalised to a geometric mean of β2-microglobulin (β2M) and β-actin mRNA expression. Differences in gene expression were determined using the 2^−ΔΔ*C*_T_^ method, and reported as fold change (relative to VEH).

**Table 1:** Primer sequences for gene expression

Gene	Primer oligo-sequence (5′-3′)
*Pgc-1α* fwd	AAGTGTGGAACTCTCTGGAACTG
*Pgc-1α* rev	GGGTTATCTTGGTTGGCTTTATG
*Tfam* fwd	CACCCAGATGCAAAACTTTCAG
*Tfam* rev	CTGCTCTTTATACTTGCTCACAG
*Cox4* fwd	ACTACCCCTTGCCTGATGTG
*Cox4* rev	GCCCACAACTGTCTTCCATT
*Nrf1* fwd	AATGTCCGCAGTGATGTCC
*Nrf1* rev	GCCTGAGTTTGTGTTTGCTG
*Nfe2l2* fwd	TGAAGTTCGCATTTTGATGGC
*Nfe2l2* rev	CTTTGGTCCTGGCATCTCTAC
*Sod1* fwd	GTGATTGGGATTGCGCAGTA
*Sod1* rev	TGGTTTGAGGGTAGCAGATGAGT
*Sod2* fwd	TTAACGCGCAGATCATGCA
*Sod2* rev	GGTGGCGTTGAGATTGTTCA
*Cat* fwd	TGAGAAGCCTAAGAACGCAATTC
*Cat* rev	CCCTTCGCAGCCATGTG
*β2M* fwd	CTGCTACGTAACACAGTTCCACCC
*β2M* rev	CATGATGCTTGATCACATGTCTCG
*β-actin* fwd	CACTGTCGAGTCGCGTCC
*β-actin* rev	TCATCCATGGCGAACTGGTG

### Protein extraction

Frozen tissue was weighed and roughly minced in a ten-fold volume of ice-cold RIPA lysis buffer, supplemented with protease inhibitor cocktail (cOmplete mini EDTA-free protease inhibitor, Roche, Mannheim, Germany) and 2 mM PMSF. Minced tissue samples were then mechanically homogenised for 1 min at an oscillation frequency of 30 Hz in a Tissue Lyser II (Qiagen, Dusseldorf, Germany). Total protein concentration was determined using a BCA-protein kit (Pierce BCA Protein Assay Kit; Thermo Fisher Scientific #23225, Rockford, IL, U.S.A.) with BSA as a standard.

### Citrate synthase activity

Citrate synthase activity was determined from protein extracts by measuring the change in absorbance at 412 nm in buffer containing 50 mM Tris/HCl (pH 8.0), 0.2 mM 5,5′-dithiobis-2-nitrobenzoic acid (DTNB), 0.1 mM acetyl-coenzyme A and 0.5 mM oxaloacetate. Samples were analysed in triplicate in 96-well microtitre plates using a Synergy 2 plate reader (BioTek Instruments Inc., Winooski, VY, U.S.A.). The slope of a linear period corresponding to at least 3 min of steady-state CS activity was determined, and the path length of the reaction measured in order to calculate CS activity using the Beer–Lambert law with an extinction coefficient of 13.7 U/mM/cm for the formation of TNB^2−^ ions. Enzyme activities are presented as enzyme units per mg protein.

### Immunoblotting

SDS/PAGE and semi-dry transfer were used to resolve and transfer 25–30 µg protein suspended in 1× Laemmli buffer (0.5 M Tris/HCl, pH 6.8, 800 mM 2-mercaptoethanol, 2 mM EGTA, 10% glycerol, 2% SDS, 0.25% Bromophenol Blue) to a nitrocellulose (catalase, SOD2 and p110α expression) or PVDF (OXPHOS proteins) membrane. Following 2 h blocking in 2% fish gelatine in TBST, membranes were incubated overnight at 4°C in primary antibodies diluted in TBST with 3% w/v BSA. Following removal of primary antibody and washing in TBST, membranes were incubated for 1–2 h in HRP-conjugated secondary antibody diluted in TBST with 2% fish gelatine. Images of proteins bands were captured using a ChemiDoc™ MP Imaging System (Bio-Rad Laboratories) in the presence of chemiluminescent substrate (Clarity Western Substrate, Bio-Rad Laboratories), and bands quantified using ImageLab 5.1 software (Bio-Rad Laboratories). Equal protein loading was confirmed by probing for GAPDH or β-actin as indicated. In the case of comparing p110α expression between liver and skeletal muscle, amido black staining was quantified to confirm equal total protein loaded. All antibody details are found in Table 2.

**Table 2:** Antibody details for immunoblotting

Target	Product code (supplier)	Lot number	Dilution used
Total Oxphos	Ab110413 (Abcam)	K2342	1:2000
Catalase	Ab16731 (Abcam)	GR319245-1	1:2000 (liver) 1:500 (skeletal muscle)
SOD2	Ab13533 (Abcam)	GR67500-59	1:5000
PI3K p110α	4249 (Cell Signaling Technology)	9	1:1000
GAPDH	Ab9485 (Abcam)	GR3243682-3	1:5000
α-Tubulin	T6074 (Sigma–Aldrich)		1:5000
Rabbit IgG	G21234 (Thermo Fisher)	2087715	1:10000
Mouse IgG	G21040 (Thermo Fisher)	2043839	1:10000

### Tissue GSH/GSSG and H_2_O_2_ content

H_2_O_2_ and glutathione levels were measured essentially as described previously [27]. To measure H_2_O_2_, tissue was mechanically homogenised in ice-cold lysis buffer (50 mM Tris pH 7.4, 50 mM sodium phosphate pH 7.4, 5 mM N-ethylmaleimide, 5 mM iodoacetamide, 12.5 mM amino-1,2,4-triazole, 0.035% sodium azide, 100 µM PMSF, 5 µM Amplex Red and 1 U/ml HRP), incubated in dark at room temperature for 10 min, clarified by centrifugation (10000×***g***, 2 min) at room temperature, and Amplex Red fluorescence was measured on a BioTek plate reader (BioTek, Winooski, VT), at an excitation wavelength of 545 nm and an emission wavelength of 590 nm every minute for 10 min. Fluorescence signal at 10 min was normalised to corresponding protein content. For total and oxidised glutathione assessment, snap-frozen tissue was mechanically homogenised in ice-cold buffer (50 mM HEPES pH 7.4, 10 mM EDTA, 1 mM EGTA, 100 mM sodium fluoride, 50 mM sodium pyrophosphate, 1 µg/ml pepstatin A, 5 µg/ml leupeptin, 5 µg/ml aprotinin, 1 mM benzamadine, 1 mM PMSF, and 5% [w/v] 3-mercaptopropionic acid), with 3 mM methyl-2-vinylpyridinium triflate for GSSG samples only. Following centrifugation, total and oxidised glutathione levels were measured in the supernatants using a BIOXYTECH GSH/GSSG-412 assay kit (Oxis International, Inc.), and normalised to protein content. GSH:GSSG ratios were determined as per the manufacturer’s instructions.

### Cell culture

To assess the effects of BYL-719 on myocytes and hepatocytes *in vitro*, C2C12 myoblasts and HEPA 1-6 hepatocytes (both from ATCC) were grown in DMEM containing 4.5 g/l and 1 g/l d-glucose (respectively), supplemented with 10% fetal bovine serum and 1% penicillin/streptomycin. C2C12 myoblasts were grown to 80% confluence, then differentiated into myotubes for 5 days using DMEM supplemented with 2% horse serum and 1% penicillin/streptomycin. Prior to experiments, C2C12 myotubes and HEPA 1-6 hepatocytes were serum-starved in DMEM containing 0.1% BSA and 1% penicillin/streptomycin for 3 h. Cells were then treated with 0–5 μM BYL-719 dissolved in DMSO for 1 h before stimulation with either 10 nM insulin or PBS as a control. Ten minutes after treatment, medium was removed, cells were washed with PBS, then lysed in RIPA lysis buffer.

### Statistical analyses

Statistical analyses were performed using Prism 8.0.0 (GraphPad Software Incorporated, California, U.S.A.), with statistical significance determined as *P*≤0.05. Unless otherwise specified, results are presented as mean ± SEM. Statistical outliers were determined by Grubbs test and removed prior to analysis. Change in body weight over time was analysed by linear regression of the change in weight from baseline measurement for each mouse. Blood glucose and plasma insulin were analysed by two-way repeated-measures ANOVA, with main effects of fasted vs fed, and VEH vs BYL. Mitochondrial respiration was analysed by two-way repeated-measures ANOVA for a difference between respiratory states and VEH vs BYL. Gene and protein expression were analysed using repeated *t* tests with Sidak post-hoc correction for multiple comparisons. Tissue H_2_O_2_ content and emission, and tissue GSH and GSH:GSSG were analysed by independent sample *t* test. In cell culture experiments, the effect of different doses of BYL-719 on Akt phosphorylation was determined by two-way ANOVA, with post-hoc comparison of each BYL-719 dose with insulin stimulation using Sidak correction for multiple comparisons.

## Results

### Oral consumption of BYL-719 results in dose-dependent hyperglycemia and hyperinsulinemia

**Figure 1:**
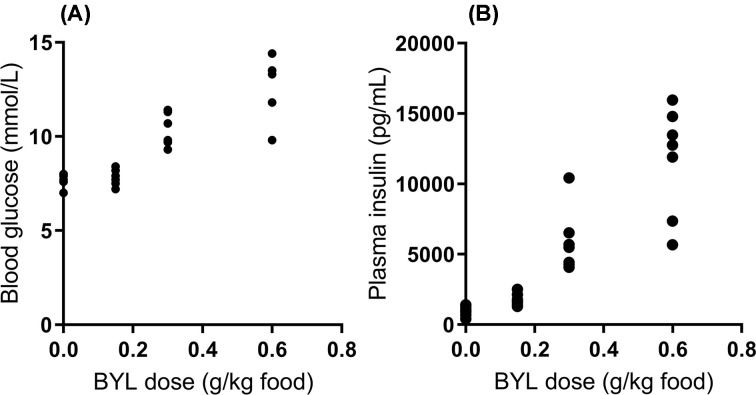
Incorporation of BYL-719 into diet increases fed blood glucose and plasma insulin Blood glucose (**A**) and plasma insulin (**B**) following 7 days of *ad libitum* access to chow containing different doses of BYL-719. Data are individual values.

To determine the effectiveness of BYL-719 consumed in diet to acutely impair glucose homoeostasis, a marker of PI3K inhibition in this context, blood glucose (Figure 1A) and plasma insulin (Figure 1B) were measured in a fed state 7 days after consuming BYL-719 in the diet. Consistent with other reports on BYL-719 [28,29], we observed a dose-dependent increase in plasma insulin and blood glucose. For all the following experiments, the dose of 0.3 g/kg of BYL-719 was used to induce a mild elevation in blood glucose levels.

### Short-term BYL-719 treatment increased hepatic markers of mitochondrial biogenesis in male but not female mice

**Figure 2:**
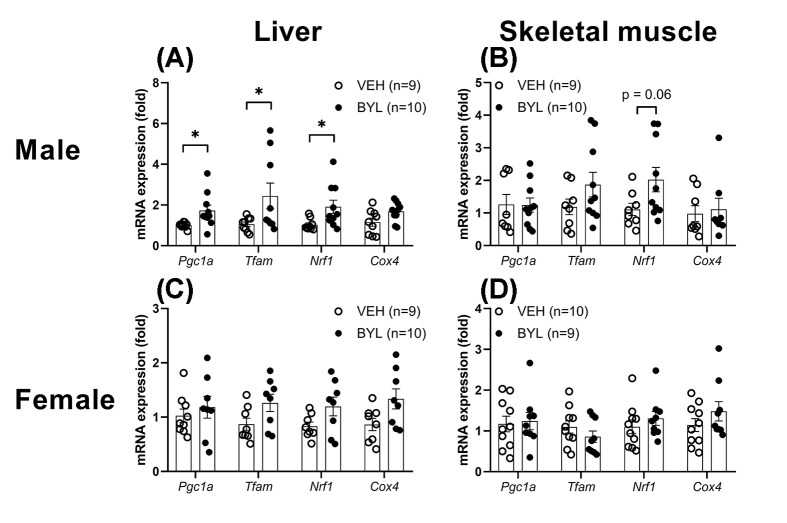
Acute effect of BYL-719 diet on gene markers of mitochondrial biogenesis Expression of mitochondrial biogenesis markers in male (**A,B**) and female (**C,D**) liver and skeletal muscle (gastrocnemius) following 72 h of *ad libitum* access to a diet containing BYL-719 (BYL) or vehicle (VEH). Data are individual values (circles) with mean ± SE, * = *P*<0.05 by *t* test.

Genetic inhibition of PI3K has previously been shown to enhance the expression mitochondrial biogenesis markers [17], therefore we first determined whether short-term (72 h) treatment with BYL-719 altered the mRNA levels of *Pgc1a, Tfam, Nrf1* and *Cox4* mRNA in the liver and muscle of VEH or BYL-719 treated mice. Expression of *Pgc1a, Tfam, Nrf1* was higher in the livers of BYL-719 male mice (Figure 2A), however, BYL-719 did not affect mitochondrial biogenesis gene markers in liver of female mice, or muscle of either sex (Figure 2B–D).

### Six weeks of BYL-719 treatment does not affect liver or muscle mitochondrial function of middle-aged mice

**Figure 3:**
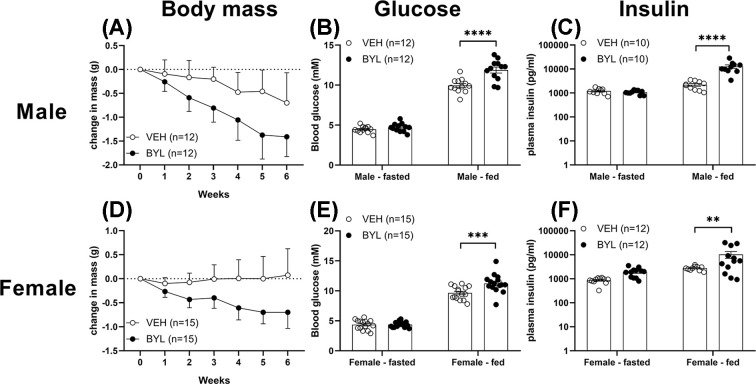
Effects of 6 weeks of BYL-719 diet on body mass, blood glucose and plasma insulin Body mass (**A,D**), blood glucose (**B,E**) and plasma insulin (**C,F**) of male (A–C) and female (D–F) mice following 6 weeks on a diet containing BYL-719 (BYL) or vehicle (VEH). Log scale is used for plasma insulin data. Data are individual values (circles) with mean ± SE, ** = *P*<0.01, *** = *P*<0.001, **** = *P*<0.0001, by two-way ANOVA.

**Figure 4:**
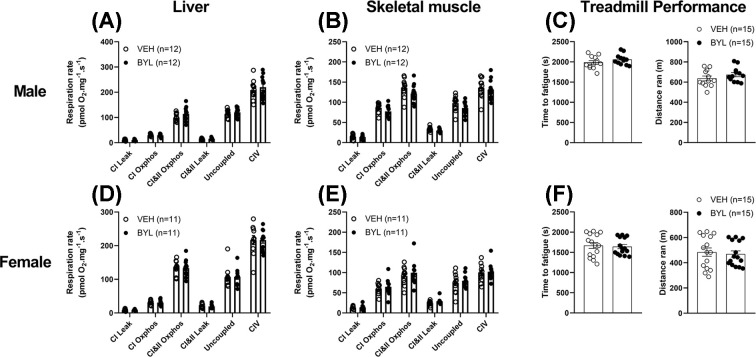
Effects of 6 weeks of BYL-719 diet on mitochondrial respiration and treadmill performance Mitochondrial respiration from liver (**A,D**) and skeletal muscle (**B,E**) and treadmill performance (**C,F**) are unchanged in males and females after 6 weeks on a diet containing BYL-719 (BYL) or vehicle (VEH). Data are individual values (circles) with mean ± SE.

Having established that short-term BYL-719 treatment increases hepatic mitochondrial biogenesis mRNA expression, we next sought to determine whether prolonged (6-week) treatment of middle-aged mice can enhance liver or muscle mitochondrial content or function. Over 6 weeks of BYL-719 treatment, body weight tended to decrease in both male and female BYL-719 mice (Figure 3A,D; *P*=0.1 in males, *P*=0.08 in females), and after 6 weeks, both males and female mice had elevated fed, but not fasted, blood glucose and plasma insulin (Figure 3B,C,E,F). Though males consumed more food than females, there was no difference in food intake between VEH and BYL groups (Supplementary Figure S1). Despite BYL-719 mildly impairing glucose homoeostasis, we did not find any differences in skeletal muscle or liver mitochondrial oxygen consumption rate between VEH and BYL groups of male or female mice, in any respiration state tested (CI Leak and Oxphos, CI and II Leak and Oxphos, Uncoupled, or CIV; Figure 4A,B,D,E). Consistent with BYL-719 not increasing maximal skeletal muscle mitochondrial respiration capacity, BYL-719 treatment had no effect on whole-body endurance capacity as assessed by time/distance to fatigue using an incremental treadmill test to exhaustion (Figure 4C,F).

**Figure 5:**
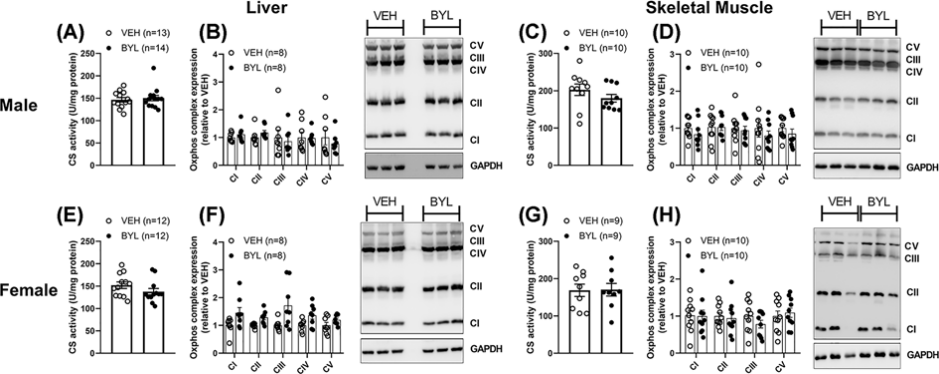
Effects of 6 weeks of BYL-719 diet on markers of mitochondrial content Six weeks of BYL-719 does not affect citrate synthase activity (**A,C,E,G**) or oxphos protein content (**B,D,F,H**) in liver or skeletal muscle of both male (**A–D**) and female (**E–H**) mice. Complex IV was not consistently detected in female skeletal muscle (**H**), and was therefore not quantified. Data are individual values (circles) with mean ± SE.

**Figure 6:**
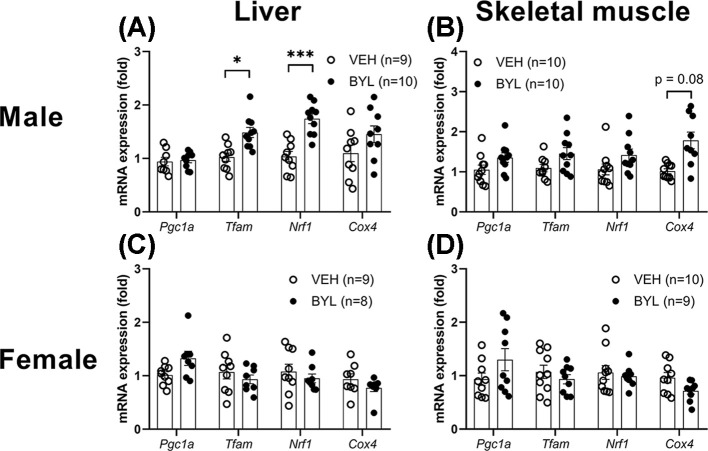
Effects of 6 weeks of BYL-719 diet on gene markers of mitochondrial biogenesis Expression of mitochondrial biogenesis markers in male (**A,B**) and female (**C,D**) liver and skeletal muscle (gastrocnemius) following 6 weeks of access to a diet containing BYL-719 (BYL) or vehicle (VEH). Data are individual values (circles) with mean ± SE, * = *P*<0.05, *** = *P*<0.001, by *t* test.

To determine if the lack of effect of BYL-719 on mitochondrial respiration could be attributed to differences in mitochondrial content, we next measured citrate synthase activity from both liver and skeletal muscle, as a proxy of mitochondrial density (2), and the expression of oxphos-related proteins. Neither citrate synthase activity nor oxphos protein expression was different in liver or skeletal muscle of males or females following BYL-719 treatment (Figure 5). Consistent with observations made at 72 h, after 6 weeks of BYL-719 treatment, male BYL-719 mice had increased markers of mitochondrial biogenesis in their livers (*Tfam, Nrf1*) but not muscle (Figure 6A,B), and no effect of BYL-719 treatment was seen on mitochondrial biogenesis markers in liver and muscle of female VEH and BYL (Figure 6C,D).

### BYL-719 treatment alters oxidative balance in the liver of middle-aged male but not middle-aged female mice

**Figure 7:**
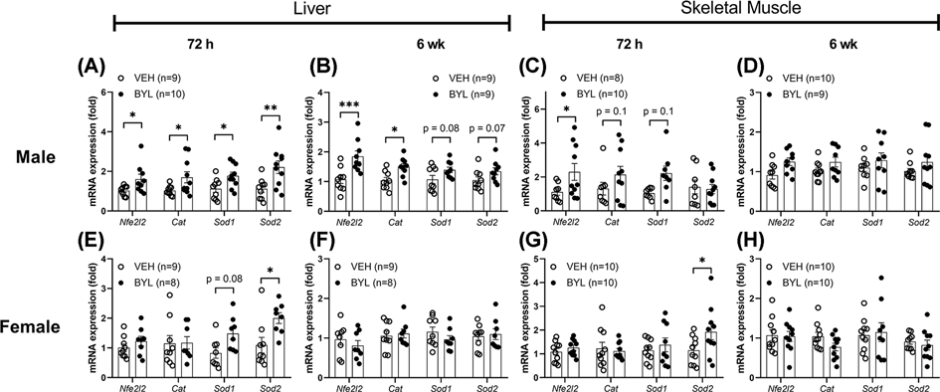
Effects of 72 h and 6 weeks of BYL-719 diet on gene markers of antioxidant defence Expression of antioxidant markers in male (**A–D**) and female (**E–H**) liver and skeletal muscle after 72 h and 6 weeks on a diet containing BYL-719 (BYL) or vehicle (VEH). Data are individual values (circles) with mean ± SE, * = *P*<0.05, ** = *P*<0.01, *** = *P*<0.001, by *t* test.

Previous studies have suggested that inhibition of insulin signalling promotes mitochondrial ROS production, and this drives cellular adaptations that extend the lifespan of model organisms [20,21,30,31]. Therefore, we next determined the effect of BYL-719 treatment on antioxidant responses in liver and skeletal muscle. After 72 h of BYL-719 treatment, mRNA expression of *Nfe2l2, Cat, Sod1* and *Sod2* were increased in liver of males (Figure 7A), however only *Sod2* was increased in liver of females (Figure 7E). *Nfe2l2* was also increased in skeletal muscle of males (Figure 7C), and *Sod2* increased in skeletal muscle of females (Figure 7G). However, after 6 weeks of treatment, female VEH and BYL-treated mice had similar levels of antioxidant genes in their liver and muscle (Figure 7F,H), while *Nfe2l2* and *Cat* reminded higher, and *Sod1* and *Sod2* tended to be higher in liver, but not muscle, of male BYL-treated mice (Figure 7B,D).

**Figure 8:**
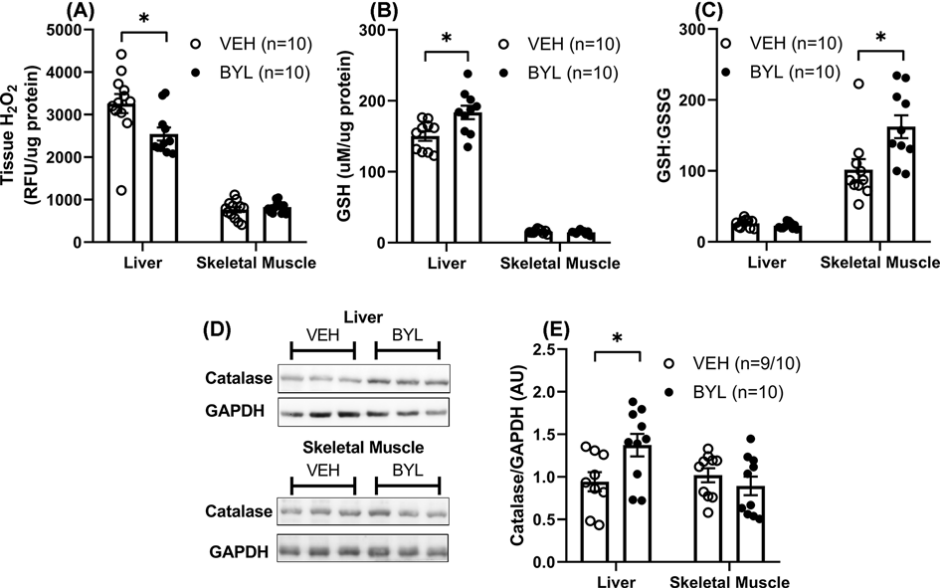
Markers of tissue oxidative stress in male mice after 6 weeks of BYL-719 diet Tissue H_2_O_2_ content (**A**), GSH content (**B**), GSH:GSSG ratio (**C**), catalase protein expression (**D,E**) in liver and skeletal muscle of male mice after 6 weeks on BYL-719 (BYL) or VEH diet. Data are individual values (circles) with mean ± SE, * = *P*<0.05 by *t* test.

Since expression levels of genes in antioxidant pathways changed primarily in males, we further investigated oxidative balance in liver and muscle from these mice. Consistent with increased catalase gene expression, the BYL group had lower H_2_O_2_ levels, as assessed by Amplex Red fluorescence (Figure 8A), and elevated liver GSH content (Figure 8B) and catalase protein expression in BYL mice (Figure 8D,E), relative to VEH. The ratio of reduced to oxidised glutathione (GSH:GSSG ratio), an index of cellular redox state, was greater in muscle, but not liver, of BYL males relative to VEH (Figure 8C). However, his does not appear to be the result of differences in mitochondrial ROS flux, as mitochondrial H_2_O_2_ emission from skeletal muscle was not different between VEH and BYL groups (Supplementary Figure S2), and SOD2 protein content was unchanged in BYL mice in either liver or skeletal muscle (Supplementary Figure S3).

### Expression of p110α is greater in liver than muscle, and hepatocytes are more sensitive to BYL-719 than myotubes

**Figure 9:**
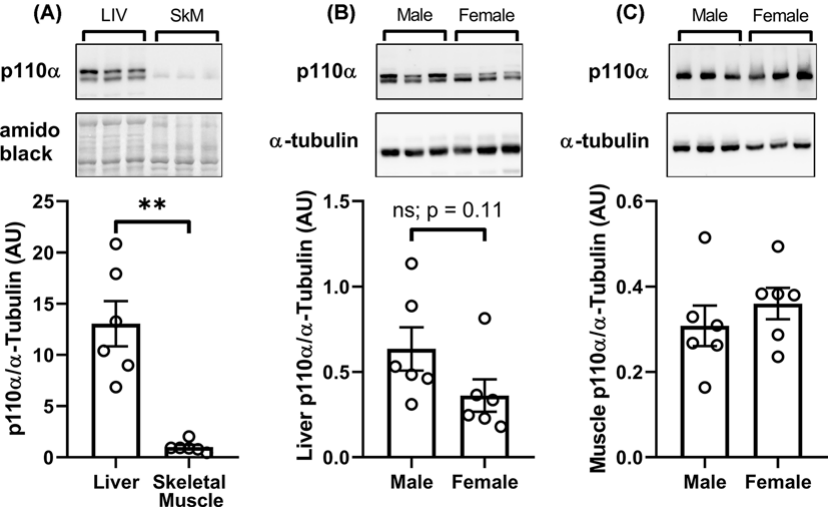
Liver expression of p110α is greater than skeletal muscle, and p110α is similarly expressed in the liver and muscle of male and female mice Expression of p110α in liver and skeletal muscle (**A**), and p110α expression in males and females liver (**B**) and skeletal muscle (**C**). Data are mean ± SE. ** = *P*<0.01.

**Figure 10:**
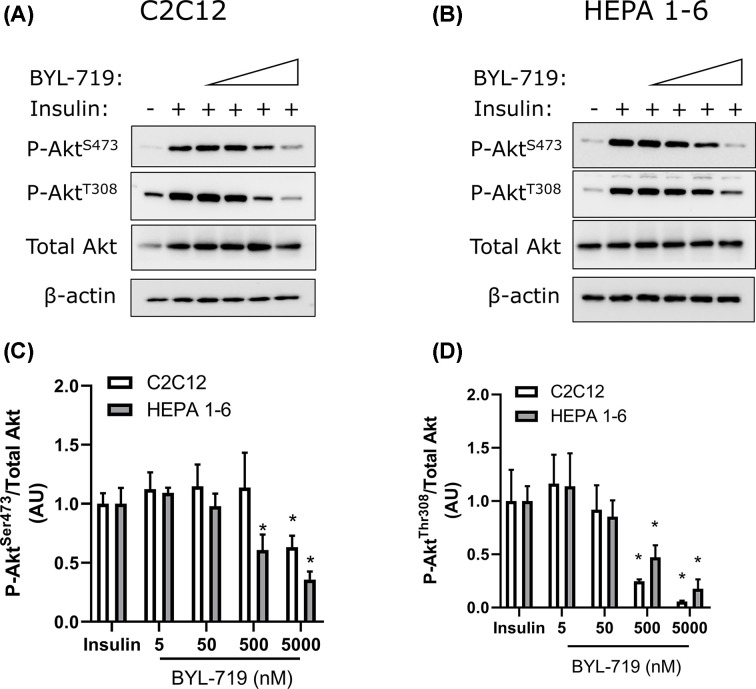
Liver Akt phosphorylation at S473 and T308 is more sensitive to BYL-719 than muscle Akt phosphorylation at S473 and T308 were determined in C2C12 myotubes and HEPA 1-6 hepatocytes following stimulation with 10 nM insulin, with different doses of BYL. Representative blots (**A,B**) and quantification (**C,D**). Data are mean ± SE, *n*=3 per condition. * = *P*<0.05 compared with insulin.

To further investigate the tissue-specific effects of BYL-719, we measured the abundance of p110α in liver and skeletal muscle from male and female mice. Abundance of p110α was greater in liver than in skeletal muscle (Figure 9A), however there were no differences in p110α expression between males and females in either tissue (Figure 9B,C). To determine whether liver and muscle cells show a similar response to BYL-719, we examined the inhibitory effect of 5–5000 nM BYL-719 on insulin-stimulated Akt phosphorylation in myoblast and hepatocyte cell lines. C2C12 myotubes were less sensitive to BYL-719-mediated suppression of insulin-induced Akt^S473^ phosphorylation compared with HEPA 1-6 hepatocytes, while insulin-induced Akt^T308^ phosphorylation was similarly suppressed by BYL-719 in myotubes and hepatocytes (Figure 10).

## Discussion

Genetic approaches that suppress the insulin-signalling cascade can alter antioxidant systems, improve mitochondrial function, and extend murine life- and health-span [10,32–34]. Here, we demonstrate that despite acutely promoting the expression of mitochondrial biogenesis genes, 6 weeks of treatment with a PI3K p110α-selective inhibitor (BYL-719) in the diet does not affect mitochondrial function or content in the liver or skeletal muscle of middle-aged mice. However, BYL-719 treatment elevated antioxidant expression in liver of males, and this was associated with reduced markers of oxidative stress. Thus, our results suggest that inhibition of PI3K p110α may enhance tissue-specific antioxidant capacity.

Mitochondria have been implicated as a mediator of many disease states, and thus preventing mitochondrial dysfunction is a potential avenue for promoting health. Although p110α inhibition did not alter mitochondrial content or function, markers of antioxidant capacity were enhanced in males. Several studies have shown that transient bursts in ROS are an important signal for cellular adaptation [35–37], and are induced by inhibiting insulin signalling or impairing glucose uptake [20,21,38]. It is therefore possible that in the early stages of treatment with BYL-719 there is an increase in ROS production, which drives the antioxidant response seen. Additionally, studies have shown in several cell lines that Akt can mediate a decrease in catalase expression through phosphorylation of Forkhead Box-O transcription factors (FOXO1 and FOXO3a) [39–41]. This presents a potential mechanism for the effects of BYL-719 in liver, whereby suppression of PI3K results in decreased Akt activity, and decreases this repression of catalase expression.

Interestingly, our data show that BYL-719 consumption increased catalase expression in liver but not in skeletal muscle, indicating tissue-specific effects. Furthermore, BYL-719 up-regulates genes for mitochondrial biogenesis in liver, but not skeletal muscle of males. Though it is likely that *in vivo* the liver would receive a greater exposure to orally consumed BYL-719 due to its role in drug metabolism, and in addition our cell line experiments suggest hepatocytes are also more sensitive to BYL-719. Furthermore, our data show that there is a much greater abundance of p110α in liver than in muscle. Both the greater abundance of p110α in liver, and greater sensitivity of hepatocytes to BYL-719 could potentially the tissue-specific effects we have seen.

Despite seeing an acute up-regulation in expression of genes relating to mitochondrial biogenesis, no difference was seen in either mitochondrial content or function in any tissues measured following 6 weeks of inhibitor treatment, and we did not see any difference in muscle mitochondrial ROS emission. This is not necessarily surprising, as increase in protein expression and eventual adaptation requires repeated transient elevations in mRNA expression [42], however, our findings are not in agreement with previous data that muscle-specific knockout of p110α increases mitochondrial oxidative capacity and free-radical production [17]. Several distinctions may explain this. Firstly, where a knockout model is a stable reduction/ablation in the expression of the enzyme, specifically in muscle, inhibitors will act transiently (particularly when taken in during feeding as in the present study) in the whole body and may not completely inhibit the target enzyme. Secondly, our study was conducted in adult mice, where we see the effects the inhibitor has after growth and development has finished. It is difficult to separate the effects of p110α knockout has over development from effects potential on an adult phenotype, particularly as the insulin/IGF signalling cascade is essential in many tissues for normal development [23,33,43–45]. The 8-month of age mice used in the present study are roughly equivalent to middle-age in humans [46,47], and in this model we see improvements in antioxidant reserves. Since the free radical theory of ageing proposes progressive damage and accumulation of mutations, perhaps PI3K p110α inhibition over a longer period and/or in older mice may lead to maintenance of mitochondrial function with ageing.

An interesting finding here is the distinct response of males and females to PI3K p110α inhibition. Though the effects seen on phenotype (body weight, blood glucose/insulin and treadmill performance) did not appear to be different between sexes, both the pattern of gene expression and tissue antioxidant status suggest that females do not up-regulate antioxidant pathways in response to p110α inhibition to the same extent as males. Consistent with this observation, male mice with a heterozygous mutation that renders the p110α subunit catalytically inactive show a more pronounced lifespan extension [11], and this may be the result of the mutant allele leading to sex-specific effects on systemic insulin, leptin and adiponectin levels [48]. Furthermore, our data suggest that liver is the more responsive tissue to BYL, and there is no difference in p110α expression between males and females, suggesting that the sex-specificity shown by our data is not the simply the result of a difference in expression of p110α.

Following 6 weeks of treatment higher fed blood glucose and plasma insulin levels were evident in the BYL-719 treated groups, and a trend towards decreased body weight was seen with BYL-719 treatment without a difference in food intake. We have previously shown that p110α inhibitors can prevent weight gain in young (4–5 weeks old) mice during development [23], which was associated with a trend towards increased basal metabolic rate. Therefore, one potential explanation for the trend in lower body weights seen here is a role for p110α in regulating metabolic rate, which is consistent with other models of PI3K inhibition [48–50]. Another potential explanation is that p110α inhibition likely prevents glucose uptake into adipose tissue, limiting substrate for lipogenesis. This is also consistent with fat-specific loss of the insulin receptor resulting in decreased fat mass, and protection from obesity [9]. It would be interesting to see the effects of p110α inhibition an adipose tissue metabolism, in particular mitochondrial function. Taken together, our results demonstrate that 6 weeks of PI3K p110α inhibition with BYL-719 has sex- and tissue-specific effects on antioxidant levels, but does not appear to affect mitochondrial function.

## Supplementary Material

Supplementary Figures S1-S3

## Open Access

Open access for this article was enabled by the participation of University of Auckland in an all-inclusive *Read & Publish* pilot with Portland Press and the Biochemical Society under a transformative agreement with CAUL.

## Abbreviations

CCCP: Carbonyl cyanide m-chlorophenyl hydrazone
CI: complex I
CIII: complex III
CS: Citrate synthase
GAPDH: Glyceraldehyde 3-phosphate dehydrogenase
GSH: Glutathione
GSSG: Glutathione disulfide
HRP: horseradish peroxidase
IGF: Insulin-like growth factor
i.p.: Intraperitoneal injection
oxphos: oxidative phosphorylation
PI3K: phosphatidylinositol-3-kinase
PMSF: Phenylmethylsulfonyl fluoride
RIPA: Radioimmunoprecipitation assay
ROS: reactive oxygen species
SOD: superoxide dismutase
TBST: Tris-buffered saline with 0.1% Tween® 20 detergent
TMPD: Tetramethyl-p-phenylenadiamine
