# Supplementary material for: Prolonged treatment with a PI3K p110α inhibitor causes sex- and tissue-dependent changes in antioxidant content, but does not affect mitochondrial function
Source: Biosci Rep. 2020 Oct 16;40(10):BSR20201128. doi: 10.1042/BSR20201128 (PMC7569204; doi:10.1042/BSR20201128)
Supplement: Supplementary Figures S1-S3 [file BSR-2020-1128_supp.pdf]

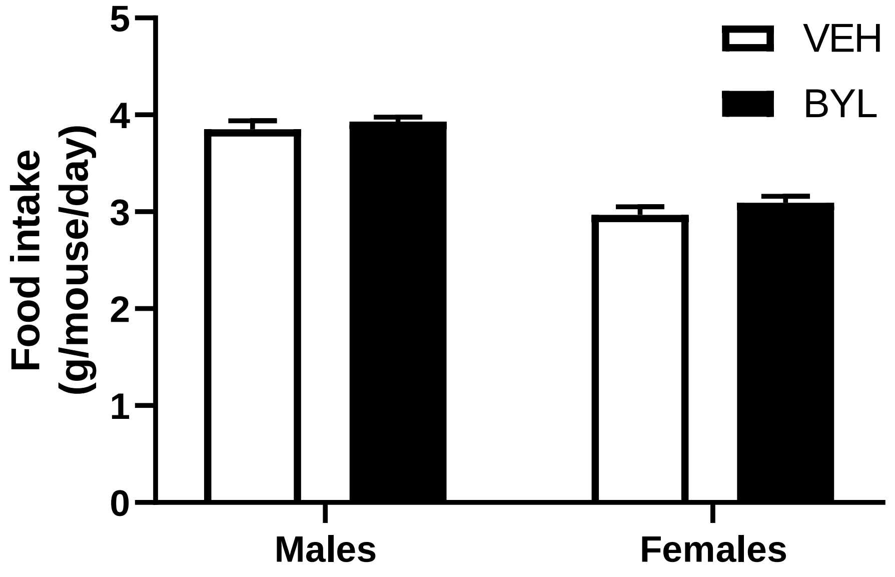

Figure S1: Food intake is not different between VEH and BYL groups.
Average food intake per mouse from VEH groups (open bars) is not different to BYL groups (filled bars). Data is mean ± SE.

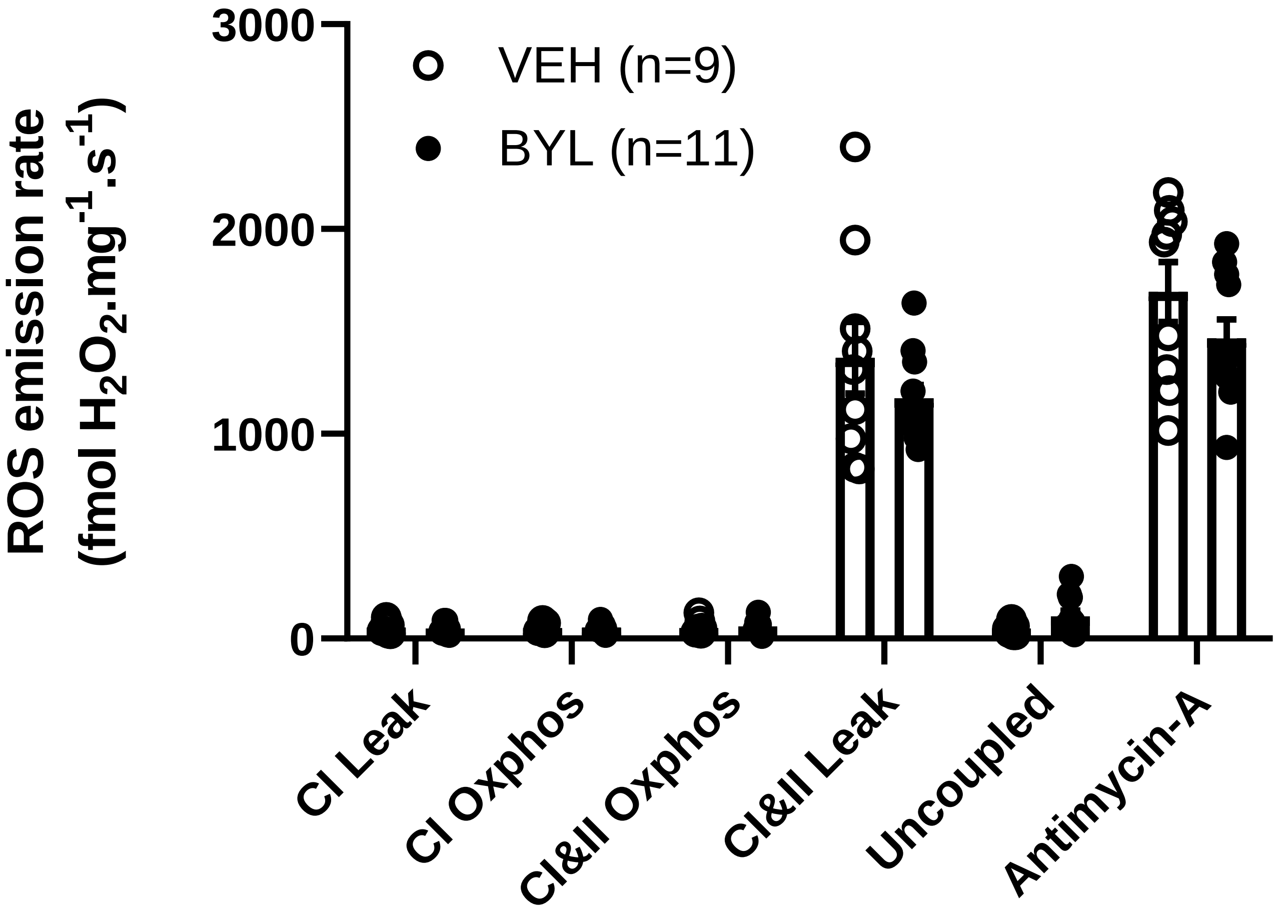

Figure S2: Skeletal muscle mitochondrial ROS emission is unchanged with BYL.
H_2O_2 release from skeletal muscle (gastrocnemius) of males is unchanged following six weeks of access to a diet containing BYL-719 (BYL) or vehicle (VEH). Data are individual values (circles) with mean ± SE.

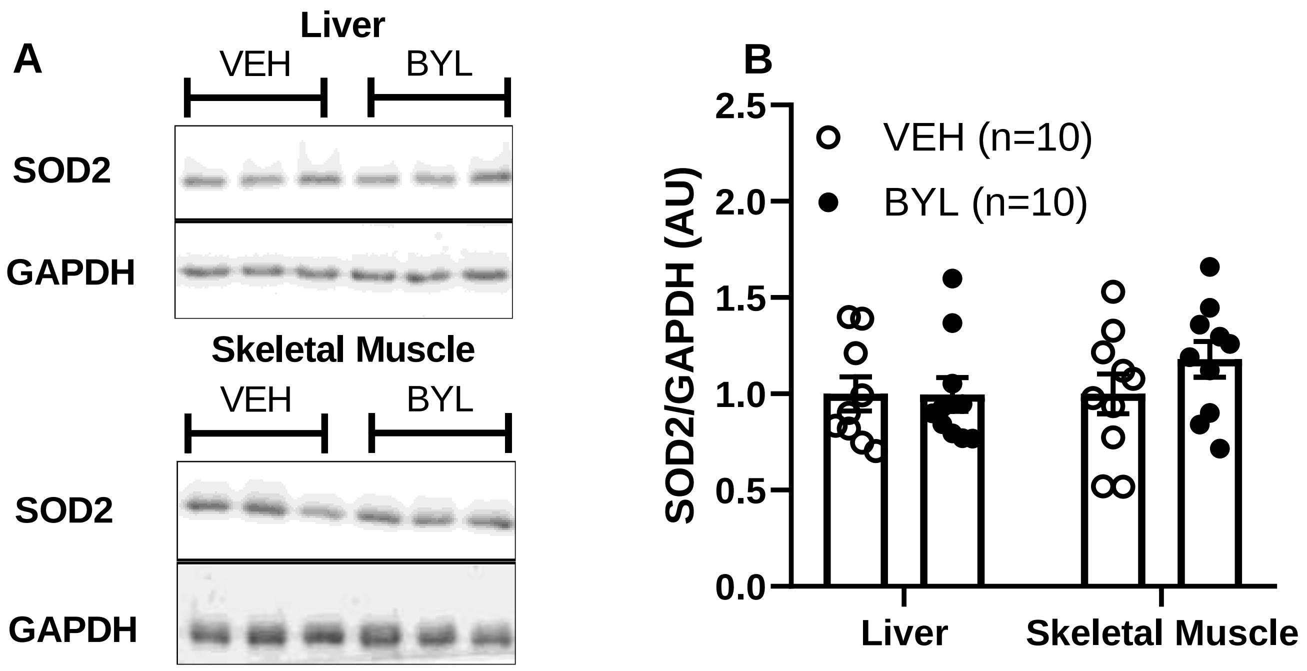

Figure S3: SOD2 expression is unchanged in liver or skeletal muscle with BYL.
SOD2 protein content is not different in liver or skeletal muscle of male mice following 6 weeks access to a diet containing BYL-719 (BYL) or vehicle (VEH). Representative blots (A) and quantification (B). Data are individual values (circles) with mean ± SE.
